# Spatial Distribution of Partner-Seeking Men Who Have Sex With Men Using Geosocial Networking Apps: Epidemiologic Study

**DOI:** 10.2196/jmir.9919

**Published:** 2018-05-31

**Authors:** Angel B Algarin, Patrick J Ward, W Jay Christian, Abby E Rudolph, Ian W Holloway, April M Young

**Affiliations:** ^1^ Robert Stempel College of Public Health & Social Work Florida International University Miami, FL United States; ^2^ Department of Epidemiology University of Kentucky Lexington, KY United States; ^3^ Department of Epidemiology Boston University School of Public Health Boston, MA United States; ^4^ Department of Social Welfare Luskin School of Public Affairs University of California Los Angeles Los Angeles, CA United States; ^5^ Center on Drug and Alcohol Research University of Kentucky Lexington, KY United States

**Keywords:** men who have sex with men, public health, mobile phone, social environment, HIV, sexually transmitted diseases

## Abstract

**Background:**

Geosocial networking apps have made sexual partner-seeking easier for men who have sex with men, raising both challenges and opportunities for human immunodeficiency virus and sexually transmitted infection prevention and research. Most studies on men who have sex with men geosocial networking app use have been conducted in large urban areas, despite research indicating similar patterns of online- and app-based sex-seeking among men who have sex with men in rural and midsize cities.

**Objective:**

The goal of our research was to examine the spatial distribution of geosocial networking app usage and characterize areas with increasing numbers of partner-seeking men who have sex with men in a midsize city in the South.

**Methods:**

Data collection points (n=62) were spaced in 2-mile increments along 9 routes (112 miles) covering the county encompassing the city. At each point, staff logged into 3 different geosocial networking apps to record the number of geosocial networking app users within a 1-mile radius. Data were collected separately during weekday daytime (9:00 AM to 4:00 PM) and weekend nighttime (8:00 PM to 12:00 AM) hours. Empirical Bayesian kriging was used to create a raster estimating the number of app users throughout the county. Raster values were summarized for each of the county's 208 Census block groups and used as the outcome measure (ie, geosocial networking app usage). Negative binomial regression and Wilcoxon signed rank sum tests were used to examine Census block group variables (eg, median income, median age) associated with geosocial networking app usage and temporal differences in app usage, respectively.

**Results:**

The number of geosocial networking app users within a 1-mile radius of the data collection points ranged from 0 to 36 during weekday daytime hours and 0 to 39 during weekend nighttime hours. In adjusted analyses, Census block group median income and percent Hispanic ethnicity were negatively associated with geosocial networking app usage for all 3 geosocial networking apps during weekday daytime and weekend nighttime hours. Population density and the presence of businesses were positively associated with geosocial networking app usage for all 3 geosocial networking apps during both times.

**Conclusions:**

In this midsize city, geosocial networking app usage was highest in areas that were more population-dense, were lower income, and had more businesses. This research is an example of how geosocial networking apps’ geospatial capabilities can be used to better understand patterns of virtual partner-seeking among men who have sex with men.

## Introduction

HIV in the United States continues to disproportionately affect men who have sex with men (MSM) despite ongoing prevention measures taken by public health officials [1]. In 2016, MSM accounted for 67% of all new HIV infections [2]. HIV and sexually transmitted infection research and intervention among MSM increasingly has focused on the social environment where risk behavior occurs [3-5], particularly as more MSM are using Web-based tools or mobile phone geosocial networking (GSN) apps (eg, Grindr, Hornet, Adam4Adam, Scruff, etc) to meet sex partners [6].

GSN apps provide information on geographic proximity between users making sexual partner seeking quick and convenient [7-10]. Research among MSM who use GSN apps to find sex partners has shown mixed results regarding the relationship between GSN app use and risky sexual practices. While some studies found no association between GSN app use for partner seeking and sexual risk behavior [11,12], some research suggests that partner seeking on GSN apps is associated with increased condomless anal intercourse [9,13,14], drug use (ie methamphetamine, Viagra, poppers, painkillers) [10,13,15], number of partners [10,13,16,17], and history of sexually transmitted infection diagnosis [15,16,18-21].

Though many studies have surveyed MSM to examine the use of GSN apps to find sex partners [10-16,18-22], relatively few studies have used the GSN apps’ geospatial capabilities to better understand geographical patterns of partner seeking among MSM. Previous research in Atlanta, Georgia, described a methodology for using the geolocation features of a GSN app as a novel approach to calculating the estimated spatial density of GSN app-using MSM. The study collected information on the closest 50 users or, when the total was less than 50, all users within a 2-mile radius of each data collection point. The data were then used to create race-stratified maps that highlighted areas of high spatial densities of MSM [23]. Research projects like the aforementioned show promise in informing geotargeted HIV prevention, treatment, and recruitment strategies. For example, geofencing, a practice widely used in mobile advertising, relies on mobile phone Global Positioning System and radio-frequency identification technology to trigger strategic HIV prevention and treatment messaging when a user enters or exits a specified area [24].

Much of the research on GSN app usage among MSM has been conducted in large urban areas [10,15,22,23,25,26], yet similar patterns of online sex seeking are reported among MSM residing in rural and midsize cities [27,28]. Smaller cities often differ from larger cities in terms of stigma [29,30] and availability of visible gay spaces [31-33]. As others have noted, research on sexual health and app-facilitated sexual behavior among MSM in midsized cities is limited [34-36]. Some research has shown that, compared with online-recruited MSM in larger cities, those in small towns were more likely to report using apps to meet long-term partners and men for sex [37]. Other studies have indicated that rural MSM experiencing hostility, stigma, and social and sexual isolation often use the internet to find sex partners [38,39]. It is important to also acknowledge that these online forums and apps can be central in facilitating positive social connectedness and friendship among MSM [40,41,42]. Taken together, these findings indicate a changing landscape of social connection and risk behavior among MSM in which technology is increasingly relevant yet not well understood, especially in settings outside of major urban centers.

This study is among the first of its kind to use the geospatial capabilities of GSN apps to provide insights on the use of 3 different GSN apps by MSM in a midsize city during weekday daytime and weekend nighttime hours, thus building upon previous research in a large urban area that focused only on 1 app and lacked detail on weekday daytime versus weekend nighttime differences [23]. The purpose of this study was to describe the spatial distribution of GSN app-using MSM in a midsize city in the South and identify geographic and demographic factors associated with areas of high numbers of GSN app users.

## Methods

### Setting

We collected information on the use of 3 different MSM GSN apps using a geographically systematic sample of points in Fayette County, Kentucky [43]. Fayette County encompasses the city of Lexington, Kentucky, and has a land area of 284 square miles and a population of 295,805 people who are predominately white (75.7%) and non-Hispanic (93.1%), with 30.7% between the ages of 18 and 34 years [43].

### Data Collection

**Figure 1:**
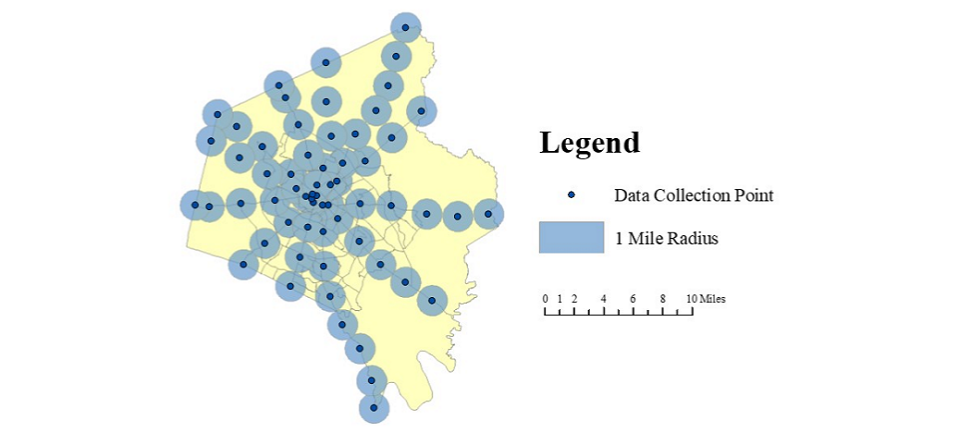
Data collection points with 1-mile coverage buffers.

The road network in the county resembles a spoke pattern in which the city is bisected by a series of main roads that extend to the county’s perimeter. The bisecting routes that offered the best county coverage were selected using Google Maps [44]. Data collection points (n=62) were spaced in 2-mile increments along 9 routes with a total driving distance of 112 miles (Figure 1).

We visited each point twice: once during weekday daytime (Monday through Friday, 9:00 am to 3:02 pm) and once during weekend nighttime (Friday or Saturday, 8:02 pm to 11:50 pm) hours. While stopped at each point (approximately 5 minutes per stop), we logged into a blank profile created for study purposes on each of the 3 GSN apps. Once we were logged into the profile, the app displayed the number of users within varying distances from the collection point. We recorded the number of users within 1 mile on each app, the time of collection, and the latitude and longitude at that collection point on a paper form and via the Fulcrum data collection app [45]. All apps used in this study have been self-described as providing a space for gay men to look for dates, friends, fun, relationships, and hookups. Apps 1 and 3 are nongroup/niche specific apps that have been described in previous research [46-49], with the latter attracting gay, bisexual, and curious men [50]. App 2 targets a subgroup identified as “bears” [47,48], who have a more traditional masculine style and acceptance of diverse body shapes [49].

According to preliminary data from interviewer-administered surveys from 57 MSM aged 18 to 35 years from Lexington (data collection ongoing), apps 1, 2, and 3 were in the top 5 most frequently named MSM GSN apps used by respondents to “meet people for sex.”

### Measures

Variables included in the regression model were derived from the 2011-2015 American Community Survey 5-year estimates [51], the 2010 Census data from the US Census Bureau [43], and the Lexington-Fayette Urban County Government Open Data Web Portal [52]. Independent variables in the model included block group population density (population count per square mile; range 3.3-15,737.5), median age (range 7.5-64.5 years), median annual household income (range $7,905-$201,429), percent white (range 10.8%-100.0%), percent Hispanic (range 0.0%-64.2%), and business zoning amounting to greater (or equal) versus less than 1% of the area of the block group (binary: 40.4% had greater or equal to 1% of the area business zoned). These variables were included in the analyses because past research on GSN app use has found that demographic variables such as age [10,15], race/ethnicity [10,15], and income [22] were associated with app use. Business zoning, the areas specified for business use by the local government, was included to capture the presence of gathering spaces (eg, restaurants, bars, shopping venues, employers).

### Analysis

#### Spatial Analyses

Of the 62 data collection locations, 61 had cell service (ie, LTE, 3G, 4G) to access apps for data collection. The point with no service was excluded from kriging analysis. We conducted spatial analyses using ArcMap version 10.3.1 (Environmental Systems Research Institute Inc) and negative binomial regression and Wilcoxon signed rank sum tests using SAS version 9.4 (SAS Institute Inc).

**Figure 2:**
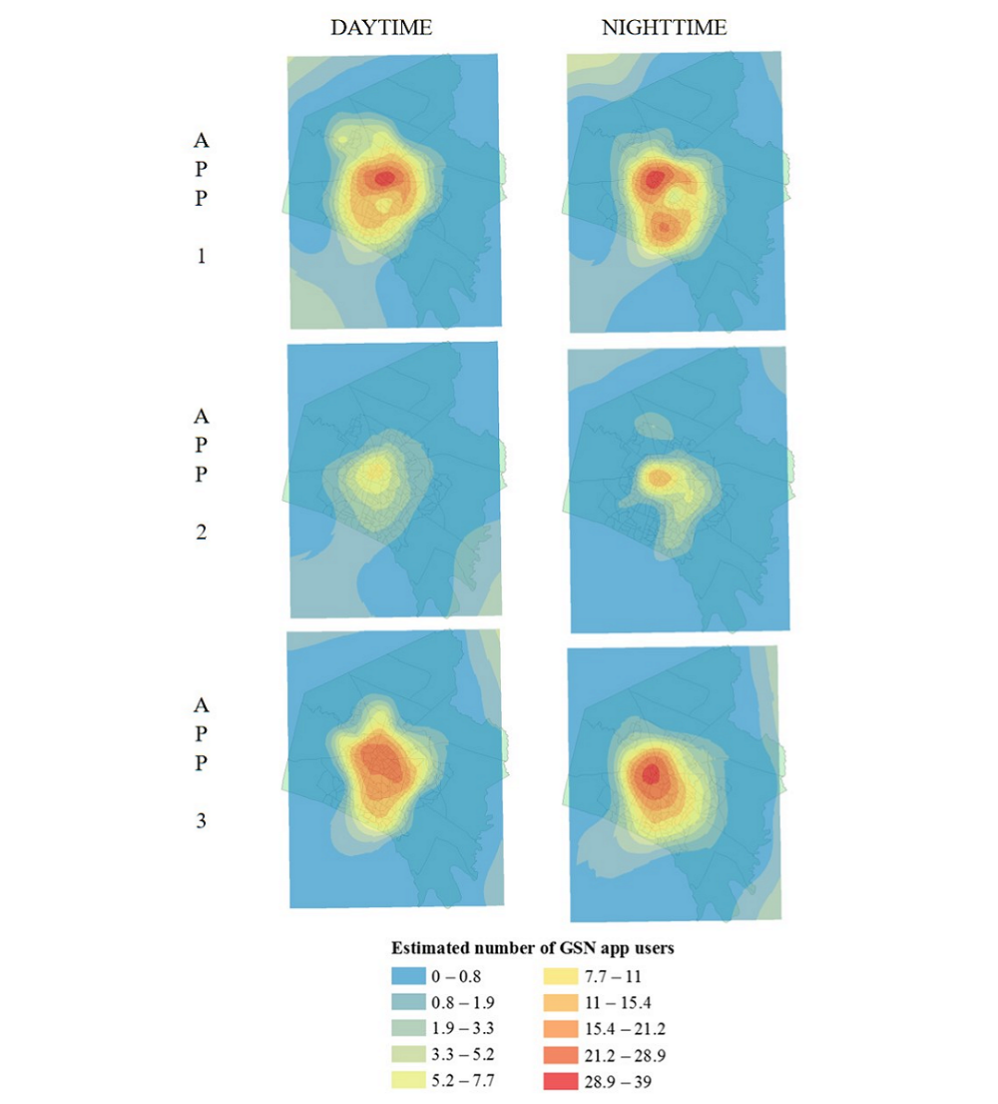
Empirical Bayesian kriging analysis of the estimated spatial distribution of geosocial networking app users by time and app.

We constructed maps to illustrate differences in the spatial distribution and number of app-using MSM across apps at different time periods. The Fayette County shapefile used in the depictions was procured from the Census Bureau [53]. We used empirical Bayesian kriging (EBK) for spatial interpolation as it predicts values for areas where data has not been collected based upon the specific values at each collected observation point and their relative proximity to other points. Past research has employed similar techniques, such as kernel smoothing, and found that kriging offered similar results [23]. The ArcMap EBK tool predicted the average number of app-using MSM for each raster grid (pixel), as show in Figure 2.

**Figure 3:**
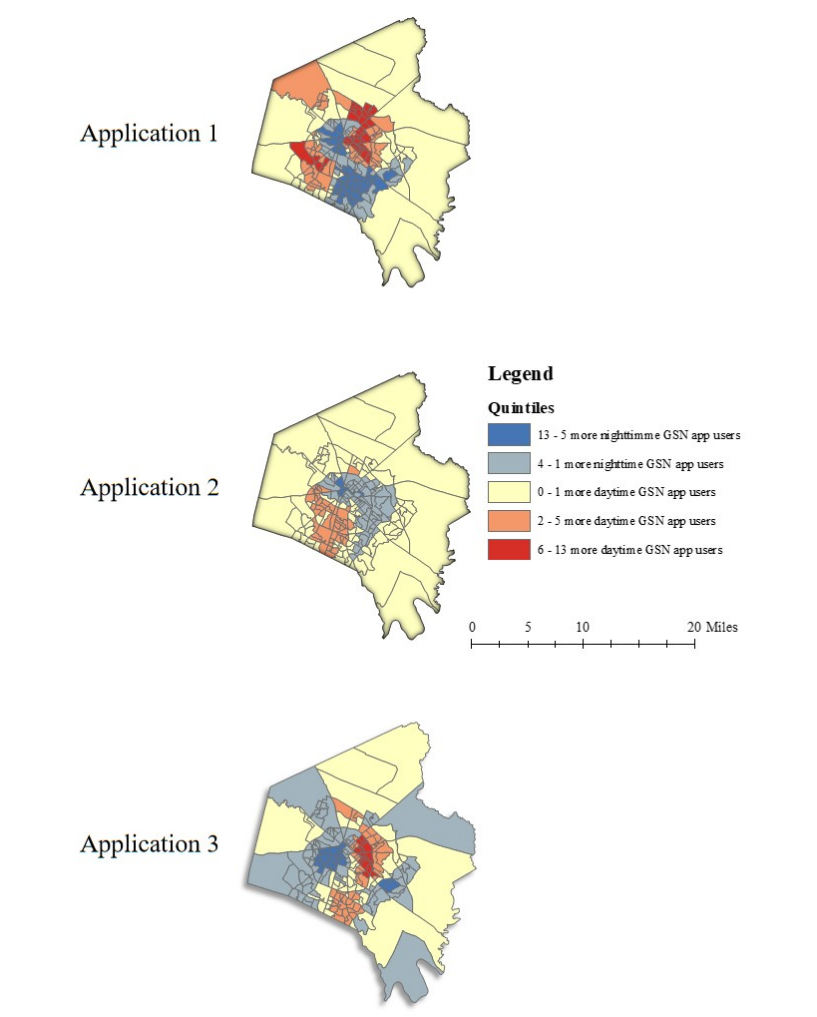
Maps displaying temporal (weekday day-weekend night) differences in the spatial distribution of app users.

We next converted the EBK raster grid cell values to points at their centroids so these values could be assigned to the Census block groups; there were from 8 to 4302 of these points in each block group, depending on area, with a median of 46. This further enabled estimation of the average number of GSN app users within each Census block group. We used this average, rounded to the nearest whole number, in each block group as the dependent variable in a negative binomial regression analysis and to calculate weekday daytime/weekend nighttime differences in the number of GSN app-using MSM by app (Figure 3).

#### Statistical Analyses

Our dependent variable was the number of app users for a particular app at a particular time period in each Census block group. Given that we examined 3 different apps, each at 2 different times, with possible duplication of users across apps and times (ie, people using more than 1 app at a time and people using the same app at different time windows), we ran 6 independent models. We used Wilcoxon signed rank sum tests to examine differences in the number of people using the app between the 3 apps and between weekday daytime and weekend nighttime data collection periods for the same app because the outcome variables were paired and nonparametric. Negative binomial regression was used to examine geographic and demographic factors associated with areas with increasing numbers of GSN app-using MSM at the Census block group level. Negative binomial regression was used because the counts were overdispersed and therefore not appropriate for a typical Poisson model. We also ran the models with the independent variables median age, median income, and population density log-transformed to try to force more linear relationships with the outcome. To test for collinearity, we ran the PROC REG collinearity diagnostic collinoint [54] to determine how related each variable was to each other in the presence of all other variables. The analysis for collinearity showed that none of the independent variables was collinear and all could be included in the same final model.

## Results

### Descriptive

The number of GSN app users within a 1-mile radius of the data collection points ranged from 0 to 36 during weekday daytime hours and 0 to 39 during weekend nighttime hours. The median number of estimated GSN app users in each Census block group in Fayette County during weekday daytime and weekend nighttime varied by app (10.0, 2.0, 10.5, and 10.0, 1.0, 9.0, respectively), but within-app comparisons revealed no significant temporal differences for any of the 3 apps (*P*=.95, .39, and .65; see Table 1).

**Table 1:** Descriptive statistics of estimated average number of geosocial networking app users per Census block group.

App and time of day		Weekday day, median (Q1,Q3)	Weekend night, median (Q1,Q3)	*P* value
App 1		10.0 (4.0,15.0)	10.0 (3.0,17.0)	.95
App 2		2.0 (1.0,4.0)	1.0 (0.0,5.0)	.39
App 3		10.5 (3.0,18.5)	9.0 (4.0,18.0)	.65
**Day**				
	App 1 vs App 2 (ref^a^)	7.0 (3.0,11.0)	—	<.001^b^
	App 2 vs App 3 (ref)	–8.0 (–14.0,–2.0)	—	<.001^b^
	App 1 vs App 3 (ref)	0.0 (–3.0,2.0)	—	.46
**Night**				
	App 1 vs App 2 (ref)	—	7.0 (2.0,14.0)	<.001^b^
	App 2 vs App 3 (ref)	—	–7.0 (–14.0,–3.0)	<.001^b^
	App 1 vs App 3 (ref)	—	–1.0 (–3.0,2.5)	.46

^a^Ref: reference group.

^b^Indicates significant difference between apps.

We created choropleth maps to display differences in the spatial distribution and number of GSN app-using MSM by time of day (Figure 3). From weekday daytime to weekend nighttime, the spatial distribution of app-using MSM varied for 2 of the 3 apps. The use of app 1 was concentrated in downtown Lexington (ie, the center of the county) during weekday daytime and weekend nighttime; however, at weekend nighttime, a second area south of the city emerged as an area of high app usage. For app 3, use was concentrated in the downtown area both during weekday daytime and weekend nighttime but use was more intensely concentrated in the downtown area at weekend nighttime.

### Block Group Regression Analysis

Fayette County comprises 208 Census block groups. Based on the dependent variable of estimated number of GSN app users in each Census block group, unadjusted negative binomial regression with each app stratified by time of collection was used to estimate crude risk ratios (Table 2). In all combinations of apps and collection times, the number of GSN app-using MSM was positively associated with population density (*P*<.001) and presence of business zoning of the block group area (*P*<.001), and negatively associated with age (*P*<.001 to *P*=.03) and median income (*P*<.001). For every 100 persons per square mile increase in population density, the number of GSN app users increased by 1.1% to 1.5%. In the presence of business zoning, the number of GSN app users increased by 106% to 188%. For every year increase in median age, the number of GSN app users decreased by 1.5% to 3%. For every $5,000 increase in median income, the number of GSN app users decreased by 5.7% to 8.9%.

Percent Hispanic ethnicity was not significantly associated with the number of GSN app-using MSM in any app or time combinations (*P*=.20 to *P*=.99). The statistical significance of the association between percent white and number of GSN app-using MSM varied between app and collection time (*P*<.001to *P*=.13); percent white was negatively associated with the number of GSN app users for app 1 and app 3 during the daytime and app 1 and app 2 during the weekend nighttime, but not for app 2 during the weekday daytime or app 3 during weekend nighttime.

The multivariable models are shown in Table 3. In all combinations of app and collection times, number of GSN app-using MSM was positively associated with population density (*P*<.001 to *P*=.045) and business zoning (*P*<.001) and negatively associated with median income (*P*<.001) and percent Hispanic (*P*<.001 to *P*=.045), adjusting for all other variables. The statistical significance of the association of median age (*P*<.001 to *P*=.46) and percent white (*P*=.02 to *P*=.77) with the outcome of interest varied between app and collection time in the final model; median age was positively associated with the number of GSN app users for app 1 during the weekday daytime but not for any other time or app. Percent white was negatively associated with the number of GSN app users for app 1 during the weekday daytime and app 2 during the weekend nighttime but not for any other time or app. In multivariable models with income, age, and population density log-transformed (data not shown in table), the results were similar except that in both of the models for app 2 and app 3, median age became statistically significant and in app 1 day and app 2 night, percent white lost statistical significance.

**Table 2:** Unadjusted analysis of the association between Census block level characteristics and number of geosocial networking app–using men who have sex with men.

Parameters		Crude incidence rate ratio (95% CI)	*P* value
**App 1: weekday day**			
	Age, year, median	0.99 (0.97-1.00)	.03
	White (%)	0.99 (0.98-0.99)	<.001
	Hispanic (%)	1.01 (1.00-1.03)	.20
	Median household income (per $5,000)	0.92 (0.91-0.94)	<.001
	Population (100 per mi^2^)	1.01 (1.01-1.02)	<.001
	Business zoning	2.24 (1.82-2.75)	<.001
**App 1: weekend night**			
	Age, year, median	0.97 (0.96-0.99)	<.001
	White (%)	0.99 (0.98-1.00)	.01
	Hispanic (%)	1.00 (0.99-1.02)	.93
	Median household income (per $5,000)	0.93 (0.92-0.95)	<.001
	Population (100 per mi^2^)	1.02 (1.01-1.02)	<.001
	Business zoning	2.06 (1.64-2.58)	<.001
**App 2: weekday day**			
	Age, year, median	0.98 (0.97-0.99)	.003
	White (%)	1.00 (0.99-1.00)	.13
	Hispanic (%)	1.00 (0.99-1.01)	.99
	Median household income (per $5,000)	0.93 (0.91-0.94)	<.001
	Population (100 per mi^2^)	1.01 (1.01-1.02)	<.001
	Business zoning	2.28 (1.89-2.73)	<.001
**App 2: weekend night**			
	Age, year, median	0.97 (0.95-0.99)	.002
	White (%)	0.99 (0.98-1.00)	.001
	Hispanic (%)	1.00 (0.97-1.08)	.65
	Median household income (per $5,000)	0.91 (0.89-0.94)	<.001
	Population (100 per mi^2^)	1.02 (1.01-1.02)	<.001
	Business zoning	2.88 (2.13-3.91)	<.001
**App 3: weekday day**			
	Age, year, median	0.98 (0.97-1.00)	.01
	White (%)	0.99 (0.99-1.00)	.049
	Hispanic (%)	1.00 (0.99-1.02)	.58
	Median household income (per $5,000)	0.94 (0.93-0.96)	<.001
	Population (100 per mi^2^)	1.01 (1.01-1.02)	<.001
	Business zoning	2.12 (1.67-2.68)	<.001
**App 3: weekend night**			
	Age, year, median	0.98 (0.97-0.99)	.001
	White (%)	1.00 (0.99-1.00)	.07
	Hispanic (%)	1.00 (0.99-1.02)	.61
	Median household income (per $5,000)	0.94 (0.93-0.96)	<.001
	Population (100 per mi^2^)	1.01 (1.01-1.02)	<.001
	Business zoning	2.26 (1.85-2.76)	<.001

**Table 3:** Multivariable analysis of the association between Census block level characteristics and number of geosocial networking app–using men who have sex with men.

Parameters		Adjusted incidence rate ratio (95% CI)	*P* value
**App 1: weekday day**			
	Age, year, median	1.03 (1.01-1.04)	<.001
	White (%)	0.99 (0.99-1.00)	.03
	Hispanic (%)	0.99 (0.98-1.00)	.01
	Median household income (per $5,000)	0.93 (0.91-0.95)	.001
	Population (100 per mi^2^)	1.00 (1.00-1.01)	.04
	Business zoning	1.69 (1.43-2.00)	.001
**App 1: weekend night**			
	Age, year, median	1.01 (0.99-1.02)	.46
	White (%)	1.00 (0.99-1.00)	.29
	Hispanic (%)	0.98 (0.97-0.99)	<.001
	Median household income (per $5,000)	0.94 (0.92-0.96)	<.001
	Population (100 per mi^2^)	1.01 (1.00-1.01)	<.001
	Business zoning	1.56 (1.28-1.92)	<.001
**App 2: weekday day**			
	Age, year, median	1.01 (1.00-1.03)	.06
	White (%)	1.00 (1.00-1.01)	.47
	Hispanic (%)	0.98 (0.97-0.99)	.003
	Median household income (per $5,000)	0.94 (0.91-0.96)	<.001
	Population (100 per mi^2^)	1.01 (1.00-1.01)	.01
	Business zoning	1.77 (1.47-2.13)	<.001
**App 2: weekend night**			
	Age, year, median	1.01 (0.99-1.03)	.36
	White (%)	0.99 (0.98-1.00)	.02
	Hispanic (%)	0.96 (0.95-0.98)	<.001
	Median household income (per $5,000)	0.93 (0.90-0.97)	<.001
	Population (100 per mi^2^)	1.01 (1.00-1.01)	.045
	Business zoning	2.22 (1.67-2.93)	<.001
**App 3: weekday day**			
	Age, year, median	1.01 (100-1.03)	.11
	White (%)	0.99 (0.99-1.01)	.64
	Hispanic (%)	0.99 (0.97-1.00)	.045
	Median household income (per $5,000)	0.95 (0.93-1.00)	<.001
	Population (100 per mi^2^)	1.01 (1.00-1.01)	.01
	Business zoning	1.77 (1.41-2.21)	<.001
**App 3: weekend night**			
	Age, year, median	1.01 (0.99-1.02)	.25
	White (%)	1.00 (1.00-1.01)	.77
	Hispanic (%)	0.99 (0.98-1.00)	.02
	Median household income (per $5,000)	0.95 (0.93-1.00)	<.001
	Population (100 per mi^2^)	1.01 (1.00-1.01)	.03
	Business zoning	1.85 (1.53-2.24)	<.001

## Discussion

### Principal Findings

This study revealed that the presence of business zoning and population density were positively associated with the number of GSN app-using MSM during both weekday daytime and weekend nighttime for all GSN apps. We also found that median income and percent of the population who were Hispanic were negatively associated with the number of GSN app-using MSM during both weekday daytime and weekend nighttime for all GSN apps, adjusting for other variables in the model. Increased app use in areas with the presence of business zoning could imply that app users may be using these apps in areas of economic activity (eg, bars, restaurants, stores) including areas that cater to a predominantly gay clientele. This could highlight an important overlap between virtual and in-person partner-seeking spaces.

By using Wilcoxon signed rank sum tests and comparing choropleth maps of the differences in the spatial density of GSN app-using MSM, we determined that the total number of users between weekday daytime and weekend nighttime was not significantly different but that specific areas within the county could be experiencing changes in the number of partner-seeking MSM between these 2 time periods. This could imply either that the same users are migrating to different areas over time, different users at different locations are logging in at different times, or a combination of these. Previous research with GSN app-using MSM indicates that more than 50% log on 5 or more times per day with significantly greater percentages logging on during evening hours [55]; however, no studies to our knowledge have tracked use by time and geographic position. This information could be informative to local health departments because instead of using mobile HIV testing units only during weekend nighttime hours at nightlife venues, these units could also be used at specified hotspots during weekday daytime hours. These data may also be able to inform more cost-efficient geotargeted and temporally targeted recruitment strategies for research. However, more research is needed to explore attitudes about the presence of HIV outreach activities such as mobile HIV testing units or research recruitment efforts near daytime hotspots such as neighborhoods and places of business.

The novel methods of data collection used in this study addressed some of the limitations of past GSN app research. For example, prior research using GSN apps for data collection largely relied on a single app [12,14,23,26,41,42,56]. Our study used 3 apps, and the variance in spatial distribution of use and number of users across apps highlights the importance of using multiple apps in future research. Popularity of GSN apps shifts over time, and some apps are more popular among specific subgroups of MSM [57]. Previous research has also aggregated geographic data across time points rather than examining daytime and nighttime use separately [23]. Our study, which examined weekday daytime and weekend nighttime use separately, revealed that spatial patterns of use may vary by time of day.

Finally, data on the use of GSN apps is also novel in the context of midsize cities as most studies to date have focused solely on larger cities [13,15-17,20,22-24,26]. Collecting data on GSN apps for MSM in midsize cities could be of increased importance due to differences in social context [29-33,38,39] and the way in which users interact with the app in contrast with urban GSN app users [37]. This is especially important given the disparities in resources for sexual and gender minority people in rural areas compared to large urban centers. Prior research has demonstrated the importance of technology in the lives of rural sexual and gender minority people in combating social isolation and homophobia in public social spaces [37-41]. In general, more research on the role of technology in fostering resilience among MSM in rural areas is needed. However, given the literature linking GSN app use and sexual risk behavior, a closer examination of how these dynamics play out in rural settings is crucial to developing targeted HIV prevention efforts for rural MSM.

### Limitations

Although the maps demonstrated substantial differences in estimated GSN app usage throughout the study area, more data collection points could have improved the accuracy of the EBK estimation. Also, our study compared differences between weekday daytime and weekend nighttime but did not include weekday nighttime hours as a sampling window. The sampling time window was large, more so for weekday daytime hours (6-hour window) than weekend nighttime hours (4-hour window); future studies may benefit from narrowing the time window of data collection; although this may require data collection occurring over more calendar time depending on personnel. Additionally, this study used a multiapp approach for data collection, but there were other GSN apps that were not used for this study. At the outset of the study, we contacted 6 app companies to notify them of our intent to do the study and dropped 3 apps due to the fact that it violated their user agreements.

In respect of app users’ privacy, we did not record any identifying information about GSN app users, making it impossible to compare demographic characteristics of users with ecological variables based on census data. This also prevented us from determining the extent to which GSN app-using MSM were using more than 1 app and whether the differences in spatial distribution were due to the temporal migration of the same users to different locations or if other app users in different locations were logging on at different times. Although the choice not to collect identifying data resulted in analytic limitations, we believe that this was an important tradeoff to make in respect of user privacy and app company policies. Ethical issues surrounding the collection and use of GSN profile data for research are complex, rapidly evolving, and warrant increased attention. In the interim, we believe it is important for researchers to communicate with GSN app companies and take steps to prevent violation of user privacy.

### Conclusion

The number of GSN app users within a 1-mile radius of the data collection points ranged from 0 to 36 during weekday daytime hours to 0 to 39 during weekend nighttime hours. In the multivariate analysis, the number of GSN app-using MSM was positively associated with business zoning and population density and negatively associated with median income and percent Hispanic residents. Methodology using geospatial data and area demographic data can lead to insights for tailored and targeted interventions to support better health outcomes in underserved populations.

## Abbreviations

EBK: empirical Bayesian kriging
GSN: geosocial networking
MSM: men who have sex with men
